# Evaluation of the Nigerian Urban Reproductive Health Initiative (NURHI) Program

**DOI:** 10.1111/sifp.12027

**Published:** 2017-06-16

**Authors:** Measurement, Learning and Evaluation Project Nigeria Team

## Abstract

Gaps remain in understanding whether family planning (FP) programs can change urban women's FP behaviors. Even less is known about what works among poor urban women. This article presents results of the impact evaluation of the Nigerian Urban Reproductive Health Initiative (NURHI). Findings are based on recently collected longitudinal data from women and facilities in six cities in Nigeria. Over the four‐year follow‐up period, there was an increase of about ten percentage points in modern method use. Impact evaluation analyses using fixed‐effects regression methods indicate that both demand‐ and supply‐side program activities increased modern method use. Radio, television, community events, and living near program‐enrolled health facilities all significantly increased modern method use or were related to a desire for no more children among all women and among poor women. Results are discussed with an eye toward the design and scale‐up of future family planning programs in urban Nigeria and elsewhere in sub‐Saharan Africa.

The benefits of increased access to and use of modern family planning (FP) are many and varied. Aside from the direct benefits of offering women and men greater control over their fertility decisions and slowing the rate of population growth, there are also indirect benefits. In particular, FP is essential to meeting the Sustainable Development Goals (SDGs) and leads to better health and well‐being (Starbird, Norton, and Marcus 2016). In Nigeria, where the maternal mortality ratio (MMR) was 576 deaths per 100,000 live births in 2013 (NPC and ICF International 2014), reducing maternal mortality is an urgent priority. Promotion of modern FP is crucial to achieving this decline. In this article we present findings from the evaluation of a family planning program in urban Nigeria.

The 2013 Nigeria Demographic and Health Survey (NDHS) found that the contraceptive prevalence rate among women in union was 15.1 percent, only a slight increase from 12.6 percent a decade earlier (NPC and ORC 2004; NPC and ICF 2014). Modern method use in 2013 was 9.8 percent nationally and 16.9 percent in urban areas, and there is wide variation in contraceptive use by geographic region, rural and urban residence, religion, education, and marital status (NPC and ICF International 2014). In the Nigerian context of low contraceptive use and high maternal mortality, there is a growing focus on increasing demand for and improving the supply of FP services, and there is growing recognition that urban areas are a critical focus for these efforts. Fifty percent of Nigeria's population was living in urban areas in 2010, and this proportion continues to rise (Fotso et al. 2011). Urban growth has created a pressing need to identify gaps in FP use and devise strategies to provide FP services to urban populations, particularly the poor.

In response to this need, the Nigerian Urban Reproductive Health Initiative (NURHI) was launched in 2010 with funding from the Bill & Melinda Gates Foundation (BMGF). At the time of launch, family planning services were available through both public and private sectors. Commodities were often lacking, however, and there was low demand for methods. In 2011, the Nigerian Government made public‐sector family planning services free for all who wish to use it and committed funds to provide supplies of commodities (Mandara 2012). By the time of the 2013 DHS, use of contraception had still not increased nationally. In the NURHI project cities, however, use was on the rise.

According to a 2011 systematic review (Mwaikambo et al. 2011), numerous demand‐ and supply‐side interventions can lead to increased knowledge of and improved attitudes toward FP. However, the interventions evaluated had not necessarily resulted in changes in behaviors such as use of contraception and use of family planning services. The evidence base regarding the efficacy of specific interventions was thin, and major gaps included a lack of evaluations of integrated, multi‐component programs and little consideration of differential impact across population subgroups including the urban poor. NURHI was designed to fill these gaps through an integrated demand‐ and supply‐side program to meet the family planning needs of urban women, particularly poor urban women. We present the results of an evaluation of the NURHI.

## NIGERIAN URBAN REPRODUCTIVE HEALTH INITIATIVE (NURHI)

NURHI was led by the Johns Hopkins Center for Communication Programs and implemented in collaboration with partners including the Association for Reproductive and Family Health and the Center for Communication Programs Nigeria. It was designed on the assumption that creating demand for FP will drive the supply of services. Demand generation for women and men focused on de‐medicalizing and demystifying family planning use, increasing understanding of and appreciation for planning one's family, supporting a person's contraceptive use, and improving knowledge and perceptions of methods in the urban contexts where the program operated. This effort was undertaken through interpersonal communication in the home, on the street, at work, in clinics, and in locations where women or men congregate. It was also done using radio and television (Krenn et al. 2014). NURHI was initially introduced in four cities: Abuja, Ibadan, Ilorin, and Kaduna. After two years of implementation, the most effective strategies were adopted in two “delayed intervention” cities: Benin City and Zaria.

## METHODS

### Data

The evaluation of NURHI was undertaken by the Measurement, Learning and Evaluation (MLE) project (also funded by BMGF) led by the Carolina Population Center at the University of North Carolina at Chapel Hill. The MLE project collected longitudinal data from women and facilities in 2010/2011 (baseline) and 2014 (endline). The evaluation focused on the aforementioned six cities. In each city at baseline, data were collected from a representative sample of women identified using a two‐stage sampling design. In the first stage, we used the 2006 Nigeria census sampling frame of enumeration areas (EA) regrouped into primary sampling units (PSUs) prior to random selection of independent samples of PSUs from each city. Given likely differences in key sampling parameters such as the expected number of women eligible for interview per household, the number of PSUs selected in a city varied from 74 in Zaria to 102 in Ibadan (MLE, NURHI, and NPC 2011). Selection at the first stage was with probability proportional to size defined in terms of the number of households in the PSU per the census frame. A household listing was conducted in each selected PSU, and 41 households were selected from each PSU by equal probability selection.

In every selected household, all women of reproductive age (15–49) were eligible for interview. All eligible women were approached by a trained interviewer and asked for their verbal consent to be interviewed. At baseline, 16,144 women (95.2 percent of those selected for interview) were successfully interviewed across the six cities. At endline, all of the baseline women who were usual residents (not visitors) of the household (16,118) were tracked and, if found, asked to be re‐interviewed. Overall, 10,672 such women were found, either at their original residence or at their new residence if they moved, and interviewed at endline for an overall follow‐up rate of 64.9 percent. For the baseline sample, the probability of selection was used to compute weights, which were equal to the inverse of the probability of selection. The endline weights included appropriate adjustments for non‐response, including correction for selective attrition. This attrition adjustment corrected for bias resulting from non‐random attrition between baseline and endline.

Women interviewed at baseline and endline are the analysis sample for examining the impact of the program on modern method use over the four‐year follow‐up period. Women who were in the two lowest wealth quintiles (poorest and poor women) at baseline are classified as “poor” for this analysis and are of particular interest; 3,954 women met this classification at baseline.

Data were collected from health facilities at baseline and endline. All public‐sector health facilities in the six cities offering reproductive health services were surveyed. The sample also included any NURHI‐enrolled private facility and any private facility mentioned by women at baseline as a source of maternal, child, FP, or HIV/AIDS services. At baseline, we collected data from 400 health facilities across the six cities, ranging from 48 health facilities in Abuja to 92 in Kaduna (MLE, NURHI, and DRMC 2011). At endline, we sought to undertake data collection in all health facilities included at baseline and in any new facilities where NURHI worked if they were not previously included. All study methods, questionnaires, and consent procedures for household, women's, and facility data collection were approved at each round by the Institutional Review Board of the University of North Carolina at Chapel Hill and the National Health Research Ethics Committee of Nigeria.

### Variables

The two key dependent variables for this analysis are use of modern contraception and desire for no more children. At baseline and endline, women were asked whether they were using a contraceptive method to delay or avoid childbearing; women who responded yes were asked what method they used. Modern methods of contraception include male and female sterilization, daily pill, IUD, implants, injectables, male and female condoms, emergency contraception, and lactational amenorrhea. Women who reported traditional method use (e.g., rhythm method, withdrawal, or folkloric methods) were coded as non‐users. At baseline and endline, women were also asked whether they wanted any (more) children. Women who responded no are coded 1; women who reported that they wanted another child or did not know are coded zero. Those who reported that they “can't get pregnant” at either survey are coded as missing and dropped from the analysis.

The key independent variables pertain to the NURHI demand‐ and supply‐side activities. The NURHI variables include both mass media (television and radio) and interpersonal communication (outreach) activities. Women were asked about exposure to NURHI mass media messages on the radio and to outreach events where NURHI discussed family planning. This was done by asking about hearing specific slogans on the radio in the local languages. In addition, cards were shown with some of the key messages being disseminated through the print media, and women were asked whether they had heard about FP at NURHI‐initiated events (e.g., at naming ceremonies and freedom ceremonies). Because NURHI activities had not begun before baseline data collection, most variables are coded zero at baseline. The exception is television exposure, which was asked at baseline and endline and was phrased in terms of exposure to any FP messages on television in the last three months. The NURHI project team reported that the only television program on family planning that was added in the study cities was the NURHI program; thus, while this variable is coded as general FP exposure via television, the increase in exposure between baseline and endline is considered to represent the NURHI television program.

Measures of supply‐side program exposure consist of characteristics of facilities that are within one kilometer of the PSU where a woman lived at the time of the survey, or within one kilometer of her residence at endline if she moved. In preliminary modeling, we examined characteristics of facilities within one kilometer, including: any facility where NURHI undertook renovations; any facility with providers trained by NURHI; provision of integrated services; and any facility that received NURHI supervision visits in the last three months. Given the lack of variation across facilities in these variables (i.e., NURHI tended to implement many activities in the same facilities), we include only one NURHI supply‐side variable in the model as a measure of overall NURHI facility‐level programs. This variable was the number of facilities where NURHI worked within one kilometer. All women are coded zero on the NURHI facility variable at baseline since these changes did not take place until the intervention began. Other general health facility characteristics not directly related to NURHI were also included in the final model and were measured at baseline and endline, namely, whether the woman lived within 1km of a facility that displayed health information, education, and communication (IEC) program materials; whether she lived within 1km of a facility with an FP outreach program; and whether any facility within 1km had a stock‐out of family planning methods in the last 30 days. All of these other supply‐side variables were factors that NURHI could have influenced, but the questions were not asked with regard to NURHI implementation and so could represent involvement by other organizations as well.

### Analysis Methods

The ideal way of producing credible estimates of the impact of NURHI would be a randomized controlled trial (RCT). However, owing to a number of constraints, including a pre‐defined set of cities where the program would be introduced and the reluctance of the program to work in a random set of neighborhoods in study cities, we were not able to implement an RCT. Therefore, our methods must take into account the fact that the program activities in the study cities were full‐coverage (i.e., all women and men were potentially exposed to program facilities and mass media activities). This creates evaluation challenges when one must rely on observational data. For instance, respondents can self‐select into “treatment” or “control” groups. In addition, capturing participation through respondent recall of exposure to various components of NURHI can introduce bias into the measurement of program impact. For example, if highly motivated individuals are both more likely to recall a specific exposure and more likely to choose to use contraception, the impact on modern contraceptive use of exposure as captured by recall could be overstated (Guilkey, Hutchinson, and Lance 2006; Hutchinson et al. 2006).

For discussion of our regression analysis and our model, see the Appendix.

While our approach allows us to estimate the impact of exposure to each program component, it does not yield a single overall measure of the impact of NURHI. In part this reflects limitations stemming from the nature of the program and our design. For instance, we lacked the funding and logistical opportunity to conduct a larger randomized control trial over many cities that might have allowed us to estimate the overall population impact of the program. Moreover, the various schemes for combining our separate marginal‐effects‐based impact estimates would not, owing to methodological and technical complexities, present a credible estimate of overall impact. Also, the true effectiveness of program activities relates to the level of impact as well as the level of program exposure (or coverage). Therefore, as discussed below, an activity might have a large impact, but if few women are exposed, the effectiveness is diminished.

## RESULTS

Table 1 shows the distribution of the characteristics of women at baseline and endline (first two columns) and response rates by demographic characteristics (last column). The distribution of the weighted baseline and endline samples is similar, though the follow‐up rates demonstrate that the women who were interviewed at endline differed significantly from those who were not re‐interviewed. For example, a significantly higher percentage of women in the oldest age groups were re‐interviewed (response rates around 75 percent) compared to women under age 35 (response rates less than 65 percent). Similarly, follow‐up rates were significantly higher among women with more children than among women with few or no children. Among women in the poorest wealth group at baseline, follow‐up rates were lower, whereas follow‐up rates in the other wealth groups ranged from 63 percent to 70 percent. Only 58 percent of never‐married women were re‐interviewed compared to 69 percent of women in union at baseline.

**Table 1:** Characteristics of women surveyed at baseline (2010) and endline (2014) and follow‐up rates from six urban sites in Nigeria

Characteristic	Baseline distribution (%) (n=16,118) 2010‡	Endline distribution (%) (n=10,672) 2014†	Percent of baseline interviewed at endline (%) (n=10,672) 2014‡
Age group			
15–19	17.7	18.0	63.5
20–24	17.7	17.0	57.7
25–29	19.6	19.6	60.5
30–34	16.5	16.7	66.0
35–39	12.9	13.0	69.4
40–44	9.0	9.2	75.5
45+	6.6	6.6	75.7***
Education			
No education/Quaranic only	11.6	11.4	71.3
Primary	14.2	14.4	67.9
Junior secondary school	10.8	10.8	63.4
Senior secondary school	38.6	39.5	64.0
Higher	23.9	23.2	62.7
Missing	0.9	0.8	57.0***
Wealth			
Poorest	18.3	16.1	59.1
Poor	19.2	19.0	63.3
Middle	20.2	20.8	65.3
Rich	20.9	22.5	69.5
Richest	21.4	21.6	66.5***
Language most commonly spoken at home			
Hausa	29.5	30.0	73.1
Yoruba	36.5	36.1	62.5
English/Pidgin English	15.1	15.1	59.6
Other languages	18.6	18.5	61.1
Missing	0.4	0.3	62.0***
City			
Abuja	12.8	13.2	63.9
Benin City	12.9	12.9	52.3
Ibadan	19.7	20.3	59.7
Ilorin	16.3	15.5	65.4
Kaduna	25.9	25.7	68.8
Zaria	12.4	12.5	78.8***
Religion			
Muslim	49.5	50.3	69.3
Christian/other Christian/other	49.5	48.6	60.6
No religion/missing	1.0	1.1	65.0***
Marital status			
In union	61.5	63.4	68.9
Separated/divorced/widowed	3.3	2.9	62.7
Never married	34.2	32.7	57.9
Missing	1.0	1.0	71.0***
Parity			
Zero	36.8	34.7	57.0
1	11.0	10.9	61.5
2	11.8	12.0	64.1
3	11.4	12.2	70.6
4	10.4	10.6	69.2
5	7.2	7.6	73.8
6	4.6	4.8	75.2
7+	7.0	7.3	82.0***
Total	100	100	64.9

NOTE: Baseline sample only includes household usual residents; visitors who were not eligible for follow‐up are not included. ‡Uses baseline weights; †Uses endline weights. ***p<0.001 indicative of significant differences between those who were found and those who were not found.

Table 2 presents the distribution of all women and poor women interviewed at baseline and endline by their contraceptive use and desire for no more children. At baseline, 71 percent of all women and 75 percent of poor women were non‐users of modern family planning. About one‐fifth of women were using modern methods and 8 percent were using traditional methods. Four years later at endline, modern method use had significantly increased to 31 percent for all women and 29 percent for poor women; traditional method use had increased to about 11 percent in both groups. Given that this is a longitudinal sample that is aging and having more children, some level of increase was expected.

**Table 2:** Contraceptive use at baseline (2010) and endline (2014) among women surveyed at both time periods in six urban sites in Nigeria

	All women		Poor† women at baseline	
	Baseline* %	Endline* %	Baseline* %	Endline* %
	2010	2014	2010	2014
Contraceptive use	(n=10,672)	(n=10,672)	(n=3,751)	(n=3,751)
Non‐user	71.2	58.4	75.5	60.2
Modern method user	21.1	30.7	18.8	29.0
Traditional method user	7.6	10.9***	5.8	10.8***
Method mix (among users)	(n=3,071)	(n=4,441)	(n=920)	(n=1,492)
Sterilization (female or male)	1.2	1.7	0.8	1.1
IUD	7.5	6.5	4.2	3.4
Implants	0.7	6.5	0.1	5.8
Oral contraceptive pill	8.0	7.6	8.4	8.6
Injectables	16.7	17.6	19.5	21.4
Condom††	29.8	22.4	29.8	17.3
Other modern method†††	9.7	11.5	13.8	15.3
Traditional method	26.5	26.2***	23.5	25.8***
Transitions in use between baseline and endline	na	(n=10,672)	na	(n=3,751)
Non‐user or traditional user both times	na	58.9	na	61.0
Non‐user or traditional user to modern user	na	20.0	na	20.2
Modern user to non‐user or traditional	na	10.4	na	10.0
Modern user both times	na	10.7	na	8.8
Desire for more children**	(n=10,197)	(n=10,197)	(n=3,575)	(n=3,575)
Wants no more	19.3	30.8***	18.0	29.6***

†Poor women are those in the two lowest wealth quintiles (poorest and poor); ††Condoms include male and female condoms (mostly male); †††Other modern methods include lactational amenorrhea, emergency contraception, diaphragm, and spermicide. na = not applicable; *Uses endline weights and presents weighted number of observations. **Drops women who report “can't get pregnant” at baseline or endline. ***All differences between baseline and endline distributions are significant at p<0.001 from the F‐test.

**Figure 1:**
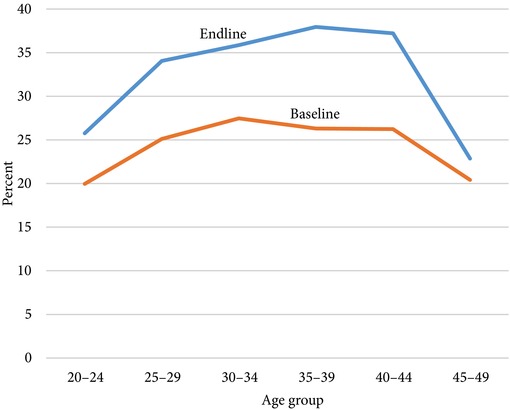
Modern contraceptive prevalence rate (percent) by woman's age at time of baseline and endline NURHI survey in six urban sites

To help interpret increases in modern contraceptive use in this longitudinal sample, Figure 1 shows the percentage of women at each age at each time point using a modern method (i.e., based on the respondent's age at the time of the baseline survey for the baseline values and the respondent's age at the time of the endline survey for endline values). Among women who were aged 20–24 at baseline (2010), about 20 percent were using a method. At endline, around 26 percent of women aged 20–24 in 2014 were using a modern method. At each age, the percentage of women using a modern method increased as demonstrated by the gap between the two bars.

Table 2 also presents the method mix among women using a method of family planning at each time period. The main methods used at baseline were condoms (30 percent) and traditional methods. Injectables were used by 17 percent of all women and 19 percent of poor women at baseline. At endline, the method mix is slightly different with a larger contribution of implants (6–7 percent at endline compared to less than 1 percent at baseline). Condom use declined while implant use increased; implants were a focus of NURHI along with IUDs.

The next part of Table 2 shows the transitions in method use between baseline and endline. The transitions for all women are similar to those for poor women. Overall, 59 percent of women were non‐users (or traditional method users) at baseline and remained non‐users at endline. Twenty percent of women were non‐users or traditional method users at baseline and transitioned to a modern method between baseline and endline. Nearly 11 percent of women were using modern methods at baseline and continued to do so at endline. Finally, 10 percent of women transitioned from modern method use at baseline to non‐use or traditional method use at endline. Given that the main methods used in this sample are spacing methods (e.g., condoms, injectables, and pills), it is not surprising that these women are transitioning to non‐use, which might reflect the desire to have a(nother) child.

At the bottom of Table 2, we give the percentage of all women and poor women who report that they do not want any (more) children at baseline and endline. At baseline, about 19 percent of women report that they do not want more children. Four years later, this figure increases significantly to 30 percent. As above, some of this increase represents natural changes over time related to women's experience of aging and giving birth in the four‐year follow‐up period.

Definitions of all program exposure measures included in this analysis are given in Table 3. Table 4 shows women's exposure to NURHI program activities at baseline and endline for all women and for poor women. With the exception of NURHI community outreach/events, poor women report slightly lower levels of exposure than all women. At baseline, about one third of all women and one quarter of poor women were exposed to FP on television in the last three months. By endline, this had increased significantly to 60 percent and 51 percent, respectively. At endline, about three quarters of both groups of women had heard a NURHI radio program. About a third of women were exposed to FP at NURHI community outreach events and through NURHI print media and billboards. Finally, about a quarter of women reported exposure to provider badges that said “Ask me about FP.”^1^

**Table 3:** Program exposure measures used in final impact analyses

Category /exposure measure	Measurement approach
**Demand**	
FP messages on TV	Heard FP messages on TV in the last three months^a^
NURHI radio	Ever heard of or listened to NURHI radio dramas Ever heard radio drama played at a meeting Ever heard a NURHI slogan on a radio drama^b^ Ever heard a NURHI radio spot/jingle^c^
NURHI community outreach	Heard FP info at any life event^d^ Heard FP info at a group or club meeting
NURHI provider badge	Saw a provider wearing a badge/button “Ask me about FP” in the last year
NURHI print media	Saw “Be Beautiful” card in the past year Saw “Be Successful” card in the past year Saw any NURHI slogan on a billboard in the past year
**Supply**	
NURHI health facility	Number of NURHI facilities within 1km of the woman
IEC program at health facility	Presence/absence of observed IEC materials in at least one health facility within 1km of the woman
FP outreach program at health facility	Presence/absence of a health facility with an FP outreach program within 1km of the woman
Stock‐out(s) of modern FP method in last 30 days	Presence/absence of a stock‐out of any modern FP method in the last 30 days at any facility within 1km of the woman

^a^General exposure to FP messages on TV is highly correlated with a composite variable of NURHI‐specific TV exposure and thus is used as a proxy measure. ^b^NURHI slogans include “Get it together”; Know, Talk, Go”; and Yoruba and Hausa local language slogans. ^c^NURHI spots/jingles include naming ceremony; hair salon/barber shop; service provider talking about FP; couple talking about FP; FP testimonial. ^d^Life events include naming ceremony, freedom ceremony, graduation; Christmas/Eid, wedding.

**Table 4:** Exposure to NURHI program activities at baseline and endline among all women and poor women

	Percentage of all women exposed to program activities		Percentage of poor† women exposed to program activities	
	Baseline	Endline	Baseline	Endline
FP messages on TV	36.1	60.1***	27.9	51.2***
NURHI radio programs	0.0	74.7	0.0	73.6
NURHI community outreach/events	0.0	32.8	0.0	34.0
NURHI provider badge	0.0	26.3	0.0	22.8
NURHI print media	0.0	37.6	0.0	34.1
NURHI health facility (within 1km)^a^	0.0	46.0	0.0	51.3
IEC program at health facility (within 1km)	70.5	70.8	74.6	76.7
FP outreach program at health facility (within 1km)	44.6	52.8**	50.5	60.5**
Stock‐out(s) in last 30 days (within 1km)	41.0	33.1**	44.5	35.8*

NOTE: All results are weighted using the endline weights; †Poor women are those in the two lowest wealth quintiles (poorest and poor); ^a^Any NURHI health facility within 1km; multivariate model uses number of NURHI facilities within 1km. Significance testing using F‐test for baseline and endline variables that were non‐zero at baseline: *p<0.05; **p<0.01; ***p<0.001.

Supply‐side variables presented in Table 4 are coded based on whether a woman lives within 1km of a facility with the specific characteristics. Since there were no NURHI‐supported facilities at baseline, this variable is coded zero at baseline. By endline, 45 percent of all women and 51 percent of poor women lived within 1km of a NURHI facility, with facilities including some combination of provider training, facility renovation, and/or commodity security. At both time points and for all women and poor women, more than 70 percent live within 1km of a facility that displayed IEC materials. At baseline 45 percent of all women and 50 percent of poor women lived within 1km of a facility that had an outreach program; by endline, this proportion increased significantly by nearly 10 percentage points in both groups. Finally, Table 4 shows an overall significant decline in stock‐outs within 1km of a woman's residence between baseline and endline from about 41 percent among all women at baseline to 33 percent at endline (a corresponding decline was observed for poor women).

Table 5 shows the marginal effects from the multivariable fixed‐effects regression analyses of modern method use. The marginal effects are the raw coefficients multiplied by 100 and can be interpreted as the average increase in the probability of using modern contraception if all members of the sample population switch from non‐exposure to exposure to the activity. For example, if all women had gone from not being exposed to being exposed to FP messages on television in the last three months, we would expect a significant increase of 2.0 percentage points in modern FP use. Further, if all women had gone from non‐exposure to exposure to the NURHI radio program, we would expect an increase of 2.9 percentage points in modern method use. This is the largest marginal effect for the set of program variables. Two other demand‐creation activities were significant: exposure to NURHI community events had a marginal effect of 2.5 and exposure to a provider badge had a marginal effect of 2.9. Significant supply‐side impacts were also observed. If all women were exposed to a FP outreach program at a health facility within 1km of where they live, modern method use would be 2.6 percentage points higher than if none were exposed to such programs.

**Table 5:** Impact of NURHI program activities on modern family planning use among women interviewed at baseline and endline

	Marginal effects of 100% program exposure on modern FP use among all women		Marginal effects of 100% program exposure on modern FP use among poor† women	
	Change in CPR due to program exposure (%)	Std. Err. (%)	Change in CPR due to program exposure (%)	Std. Err. (%)
FP messages on TV	2.0*	0.9	–0.3	1.5
NURHI radio programs	2.9**	1.1	5.1**	1.7
NURHI community outreach/events	2.5*	1.2	2.7	1.8
NURHI provider badge	2.9+	1.6	6.2*	2.9*
NURHI print media	–0.4	1.2	–1.7	2.1
Number of NURHI health facilities (within 1km)	0.9	0.7	1.0	1.0
IEC program at health facility (within 1km)	0.9	1.7	2.6	2.9
FP outreach program at health facility (within 1km)	2.6*	1.2	0.9	1.8
Stock‐out(s) in last 30 days (within 1km)	0.1	1.0	0.1	1.5
Age group				
15–19	ref		ref	
20–24	11.6***	1.5	13.1***	2.5
25–29	11.2***	2.4	10.3**	3.8
30–34	9.1**	2.9	10.5*	4.6
35–39	9.1**	3.3	10.6*	5.1
40–44	12.4***	3.9	13.0*	5.9
45+	4.1	4.2	6.1	6.3
Education				
No education/Quaranic only	ref		ref	
Primary	–0.5	1.7	0.1	2.3
Junior secondary school	1.4	2.2	5.0	3.1
Senior secondary school	0.9	2.1	4.3	2.8
Higher	5.7*	2.6	5.1	4.7
Wealth				
Poorest	0.9	1.8	8.9+	4.9
Poor	1.6	1.7	10.1*	4.8
Middle	1.6	1.6	10.6*	5.0
Rich	1.1	1.4	10.7*	5.3
Richest	ref		ref	
Language most commonly spoken at home				
Hausa	ref		ref	
Yoruba	–2.4	3.3	–1.0	6.2
English/Pidgin English	–2.3	2.4	–0.8	4.4
Other languages	–4.5+	2.4+	1.4	4.6
Religion	–0.2	2.6	–2.5	4.0
Muslim				
Christian/other Christian/other	ref		ref	
No religion/missing	–1.7	7.6	–7.6	10.8
Marital status				
In union	–12.6***	2.2	–13.6***	3.5
Separated/divorced/widowed	–20.6***	3.1	–19.3***	4.8
Never married	ref		ref	
Parity				
Zero	ref		ref	
1	9.8***	2.2	12.7***	3.6
2	17.6***	2.3	23.5***	3.8
3	24.9***	2.7	29.8***	4.1
4	30.4***	3.1	33.1***	4.8
5	36.2***	3.3	39.6***	4.9
6	37.7***	3.6	36.5***	5.5
7+	38.6***	3.6	40.3***	5.4

NOTE: All results are unweighted; †Poor women are those in the two lowest wealth quintiles (poorest and poor).

^+^p ≤ 0.10; *p ≤ 0.05; **p ≤ 0.01; ***p ≤ 0.001.

Table 5 also presents results for poor women. If all poor women went from not being exposed to being exposed to NURHI radio programs, we would expect contraceptive use to be 5.1 percentage points higher. Likewise, if all poor women went from non‐exposure to exposure to the NURHI provider badge, use would be 6.2 percentage points higher. If all poor women were exposed to NURHI community events, we would expect modern contraceptive use to be 2.7 percentage points higher. No other demand‐ and supply‐side factors were significantly related to modern method use among poor women.

The impact results presented above reflect the average change in the outcome if all women switched from being unexposed to exposed to the various program components. In reality, not all women were exposed to these components. Only about a third of women were exposed to outreach events and three quarters to the radio. In these cases, the effectiveness of NURHI activities is attenuated by the level of exposure such that the effect of radio programs needs to be multiplied by ¾ (i.e., 2.94*0.75 = 2.17) and outreach by ⅓ (2.46*0.33=0.81), lowering the overall effectiveness of the program activities. Nonetheless, the impact results represent the accepted approach to answering the basic question of which program components can and cannot influence behavior.

Table 5 also shows the effects of the demographic factors on modern contraceptive use among all women and among poor women. As expected, older, better‐educated, and higher‐parity women are more likely to use a modern method, while women in union are the least likely to do so.

Table 6 presents marginal effects from the multivariable fixed‐effects regression analyses of the desire for no more children. The marginal effects are the raw coefficients multiplied by 100 and can be interpreted as the average increase in the probability of desiring no more children if all members of the sample population switch from non‐exposure to exposure to the activity. Among all women and among poor women, we find results similar to those for modern contraceptive use. For instance, switching all women from no exposure to NURHI radio programs to full exposure would increase the proportion of women desiring no more children by 5.5 percentage points. The one variable that is significant in the unexpected direction is exposure to print media: if all women went from being unexposed to exposed to print media, there would be significantly less desire to have no more children; this may reflect women with higher parity or higher fertility desires spending more time in facilities and having greater access to this print media. Generally, the demographic variables have effects in the expected direction, with women in the prime reproductive years (ages 20–34) being least likely to report a desire for no (more) children whereas the oldest women (and the women with the most children) are the most likely to give this response.

**Table 6:** Impact of NURHI program activities on desire for no more children among women interviewed at baseline and endline

	Marginal effects of 100% program exposure on desire for no more children among all women		Marginal effects of 100% program exposure on desire for no more children among poor† women	
	Change in desire for no more children due to program exposure (%)	Std. Err. (%)	Change in desire for no more children due to program exposure (%)	Std. Err. (%)
FP messages on TV	–0.5	0.7	–0.0	1.1
NURHI radio programs	5.5***	0.8	4.6***	1.4
NURHI community outreach/events	1.3	1.0	–0.0	1.6
NURHI provider badge	3.3**	1.0	4.1*	1.8
NURHI print media	–2.1	0.9	–2.1	1.6
Number of NURHI health facilities (within 1km)	1.3**	0.5	1.3+	0.8
IEC program at health facility (within 1km)	–0.6	1.3	0.8	1.8
FP outreach program at health facility (within 1km)	1.3	1.0	2.1	1.7
Age group				
15–19	ref		ref	
20–24	–4.9***	0.6	–4.9***	0.8
25–29	–10.7***	1.2	–10.1***	1.9
30–34	–9.8***	1.7	–9.4***	2.8
35–39	–1.2	2.4	–2.9	3.7
40–44	8.5**	2.8	6.3	4.5
45+	19.7***	3.4	15.5**	5.7
Education				
No education/Quaranic only	ref		ref	
Primary	–1.2	1.9	0.7	2.4
Junior secondary school	–0.8	2.1	0.3	2.6
Senior secondary school	–2.9	2.0	–3.3	2.6
Higher	–1.2	2.1	–4.1	3.3
Wealth				
Poorest	–2.1+	1.3	–4.4	3.6
Poor	–1.6	1.1	–2.9	3.6
Middle	0.2	1.2	2.8	3.9
Rich	–1.9+	1.0	–0.9	3.9
Richest	ref		ref	
Language most commonly spoken at home				
Hausa	ref		ref	
Yoruba	–1.3	2.3	2.1	4.4
English/Pidgin English	0.2	1.9	1.3	3.6
Other languages	–0.2	2.0	3.9	3.6
Religion				
Muslim	0.4	2.0	–2.9	3.2
Christian/other Christian/other	ref		ref	
No religion/missing	–3.6	4.7	–2.5	4.0
Marital status				
In union	–1.5+	0.9	–3.5**	1.3
Separated/divorced/widowed	9.0***	2.5	4.4	3.2
Never married	ref		ref	
Parity				
Zero	ref		ref	
1	–1.6+	0.9	1.5	1.4
2	–0.6	1.1	–0.5	1.9
3	8.9***	1.7	7.1**	2.5
4	18.0***	2.3	13.7***	3.3
5	30.6***	2.8	23.5***	4.0
6	35.7***	3.2	27.7***	4.6
7+	42.9***	3.3	35.8***	4.7

NOTE: All results are unweighted; †Poor women are those in the two lowest wealth quintiles (poorest and poor).

^+^p ≤ 0.10; *p ≤ 0.05; **p ≤ 0.01; ***p ≤ 0.001.

## DISCUSSION

This study demonstrated that NURHI, a multi‐component program with both demand and supply elements that targeted urban areas of Nigeria, led to significant increases in modern contraceptive method use and declines in the desire for more children in a short period of time. Overall, we observed an increase in modern method use between baseline and endline of about 10 percentage points and increases in the desire for no more children by a similar amount. Because we used longitudinal data where the sample at endline is about four years older and has had more children and had a chance to get married, it is not surprising that method use and desire for no more children increased over time. In our multivariate fixed‐effects regression models, we partially controlled for these demographic changes, and our results suggest that a number of NURHI activities are associated with the increases in use and increased desire for no more children. In particular, we find that among all women, exposure to the various demand‐creation activities—television, radio, community outreach at key events, and the provider badge—was associated with increases in modern method use. On the supply side, living within 1km of a facility where NURHI made facility improvements, including renovations, training of providers, and commodity security, was borderline associated with increased modern method use over time. Among poor urban women at baseline, demand‐generation activities (radio and the provider badge) were significantly associated with modern method use, and community events were borderline significant. We found similar results for NURHI demand‐ and supply‐side activities on desire for no more children among all women and among poor women.

Previous research has demonstrated that multi‐channel communication initiatives lead to greater impacts than single‐channel programs (Noar et al. 2009; TAG 2015). Our findings are consistent with previous findings that radio, television, interpersonal outreach at significant life events, and FP promotion through the “Ask me about FP” badge were significantly related to modern method use and desire for no more children. Less evidence exists on multi‐component programs that incorporate both demand‐side and supply‐side activities, as was done in the NURHI program (Mwaikambo et al. 2011). Clearly in a context like urban Nigeria, there is a need to create a demand but also to ensure that high‐quality services are accessible.

This article is one of a handful to evaluate multi‐component programs for increasing family planning use in a developing country. Few prior family planning evaluations focused specifically on urban areas. Nevertheless, the article has some limitations. First, we were unable to re‐interview about one‐third of the women at endline. The women whom we could not locate tended to be younger, single, from certain cities, and in the poorest wealth group. Weights were used to adjust for the characteristics of the missing women at endline. Second, because the facility‐level activities were comprehensive, it was not possible to tease apart the impact of quality improvement or integration on modern method use. Third, the exposure variables were self‐reported, thus there may be self‐selection among those who report being exposed (or remember being exposed) as compared to those who report no exposure (or do not recall exposure). By using fixed‐effects analyses, we are able to control for some of this self‐reporting bias. Finally, the sample size and observed variation did not permit estimation of stable, credible interaction effects between program components.

This study demonstrates that even in a context like urban Nigeria, with high maternal mortality and low contraceptive use, targeted programs can lead to important changes in modern method use and fertility desires in a short period of time. Impacts were observed among all women and among urban poor women. Poor urban women have latent needs for FP that programs can address by using local radio and outreach at life events. The evidence from this impact evaluation is being used by the NURHI team to inform scale‐up and expansion of programs into other urban areas and into rural areas. The findings from this study should also be used to inform future evidence‐based family planning programs in urban and rural Nigeria and elsewhere in sub‐Saharan Africa, especially those targeting the poor.

## Footnotes

1 For differences in exposure to program activities by city, see the endline project report at: https://www.urbanreproductivehealth.org/resource/measurement-learning-evaluation-urban-reproductive-health-initiative-nigeria-2014-endline.

## Appendix

We rely on longitudinal data where each woman is observed before and after program implementation. Specifically, we apply fixed‐effects regression to the pooled samples (baseline and endline) to reduce bias associated with self‐selection, recall bias, and program targeting to underserved areas due to time‐invariant unobservables. These time‐invariant unobservables are differenced out in the estimation method. Fixed‐effects methods also control for the possibility of endogenous attrition in the endline sample to the extent that attrition is due to unobserved fixed characteristics of individuals, since these fixed characteristics are also differenced out of the estimated model as long as these time‐invariant unobservables do not have a time‐varying effect on the outcome. Fixed‐effects methods do not control for time‐varying unobservables. Since observations are closely spaced, less time‐varying unobservables (such as motivation) are likely to be the main source of residual bias. We estimated a classic fixed‐effects or first‐differences model in which changes in modern contraceptive use (and desire for no more children) depend on changes in time‐varying individual characteristics and program exposure.

In our model, *Y_i,t_* is modern contraceptive use for individual *i* at time *t* (where *t* = 0, 1 for baseline and endline, respectively) and *X_ij,t_*, *R_ik,t_*, and *P_il,t_* are, respectively, controls for a characteristic *j* (out of *J* individual characteristics included in the model) of individual *i* at time *t* (see Table 1 for relevant control variables in the model), exposure of individual *i* to non‐NURHI supply‐side variable *k* (out of three) at time *t*, and exposure of individual *i* to NURHI program exposure *l* (out of 6 NURHI program components) at time *t* (see Tables 3 and 4 for measurement and exposure to NURHI and non‐NURHI program variables). Our basic estimation model is then:

$$\begin{array}{ccc} Y_{i,1}-Y_{i,0} & = & \beta_{0}+\beta_{1}\cdot \left(X_{i1,1}-X_{i1,0}\right)+\cdots +\beta_{J}\cdot \left(X_{iJ,1}-X_{iJ,0}\right)+\varphi_{1}\cdot \left(R_{i1,1}-R_{i1,0}\right)+\cdots \\ & & +\;\;\varphi_{3}\cdot \left(R_{i3,1}-R_{i3,0}\right)+\theta_{1}\cdot \left(P_{i1,1}-P_{i1,0}\right)+\cdots +\theta_{6}\cdot \left(P_{i6,1}-P_{i6,0}\right)+\varepsilon_{i,t} \end{array}$$

We estimate this via the linear probability model. The θs thus represent the impact of the various program components as captured by the marginal effect of the program components on the probability of modern contraceptive use. Specifically, the true population regression model underlying our fixed‐effects estimator is:

$$\begin{array}{ccc} Y_{i,t} & = & \left(\beta_{0}\right)+\beta_{1}\cdot x_{i1,t}+\cdots +\beta_{J}\cdot x_{iJ,t}+\varphi_{1}\cdot R_{i1,t}+\cdots +\varphi_{3}\cdot R_{i3,t}+\theta_{1}\cdot P_{i1,t}+\cdots \\ & & +\;\;\theta_{6}\cdot P_{i6,t}+\varepsilon_{i,t} \end{array}$$

Our fixed‐effects model simply subtracts from each regressor for individual *i* at time *t* its mean over time for that regressor. However, the fixed‐effects model still estimates the coefficients from this regression. These estimates, which are reported in Tables 5 and 6, are interpretable as marginal effects. Marginal effects are the change in the probability of the outcome (e.g. use of modern family planning in Table 5) occurring when the value of each regressor (such as a program component) is switched from 0 to 1 for each member of the sample. Thus, for example, the estimates in Tables 5 and 6 indicate how the probability of the outcome would change if each person in the sample switched from being unexposed to exposed to each program component in the regression model. (A similar interpretation applies to the controls for individual characteristics.)
